# Metagenomic Cell-free DNA Sequencing for Treatment Monitoring in Sepsis

**DOI:** 10.21203/rs.3.rs-8148988/v1

**Published:** 2025-12-22

**Authors:** Iwijn De Vlaminck, Omary Mzava, Liz-Audrey Djomnang, Alexandre Cheng, Luis Gomez-Escobar, Joan Lenz, Emma Belcher, Edward Schenck

**Affiliations:** Cornell University; Cornell University; Cornell University; Cornell University; Weill Cornell Medicine; Cornell University; Cornell University; Weill Cornell Medicine

## Abstract

Sepsis is a life-threatening organ dysfunction caused by a dysregulated response to infection. Early identification of pathogens and accurate assessment of organ injury are critical for improving outcomes, but current methods are often inadequate, especially after initiation of antibiotic treatment. Metagenomic sequencing of cell-free DNA (cfDNA) offers a promising alternative, enabling simultaneous pathogen detection and tissue-of-origin profiling. Contamination, however, can limit its accuracy in low-biomass samples. Here, we apply the Sample-Intrinsic Microbial DNA Found by Tagging and Sequencing (SIFT-seq) assay, which reduces contamination and allows detection of pathogens and organ injury simultaneously. We analyzed 142 plasma specimens: 105 from sepsis patients, 103 collected after initiation of antibiotic treatment, 24 from non-sepsis ICU controls, and 13 from healthy controls. SIFT-seq identified sepsis-causing pathogens in good agreement with pre-antibiotic blood cultures, revealed elevated immune activity and organ injury in sepsis patients, and, when combined with the SOFA score in a multivariate model, improved diagnostic performance (AUC = 0.874). These findings highlight the potential of integrated cfDNA profiling to enhance sepsis diagnosis.

## INTRODUCTION

Sepsis is a life-threatening condition in which a dysregulated host response to infection leads to organ dysfunction^1^. In 2017, the World Health Organization estimated sepsis was responsible for nearly one in five global deaths^2,3^. A major challenge in managing sepsis is the objective quantification of organ damage and dysfunction, a complication of sepsisstrongly associated with mortality^4,5^. The Sequential Organ Failure Assessment (SOFA) score, the current clinical standard, requires multiple laboratory tests that are not always available at the bedside^6^ and evaluates only six organ systems, despite broader organ involvement. More comprehensive and rapid tools to measure organ dysfunction are urgently needed.

Equally important is the early and accurate identification of the causative pathogen to guide therapy and prevent further organ injury and death. Bacteremia, common in sepsis, is associated with worse outcomes^5,7^. Yet in over 30% of cases, the causative pathogen is never identified^5,7^. This uncertainty drives widespread use of broad-spectrum antimicrobials, increasing risks of microbiome disruption and antimicrobial resistance^8,9^. Blood culture, the gold standard, detects only culturable microbes and often fails after antibiotics are initiated^10,11^. Metagenomic sequencing of cfDNA offers broad-range detection, high sensitivity, and rapid turnaround^12–16^, but suffers from environmental DNA contamination which limits specificity.

To overcome this, we previously developed the SIFT-seq assay^17^, which chemically tags sample-intrinsic cfDNA by converting unmethylated cytosines through bisulfite treatment. This tagging is performed on the biofluid before DNA isolation and library preparation, allowing intrinsic cfDNA to be distinguished from contaminating DNA introduced later, since contaminant DNA retains unmethylated cytosines. In parallel, because DNA methylation patterns are cell- and tissue-specific, and cfDNA abundance reflects cell death,^15,18,19^, SIFT-seq can also reveal organ and tissue injury. Here, we applied SIFT-seq to 142 plasma samples from septic and non-septic ICU patients and healthy volunteers to evaluate its utility for pathogen detection after antibiotic initiation and for quantifying sepsis-related organ damage.

## RESULTS

### Study cohort

**Figure 1:**
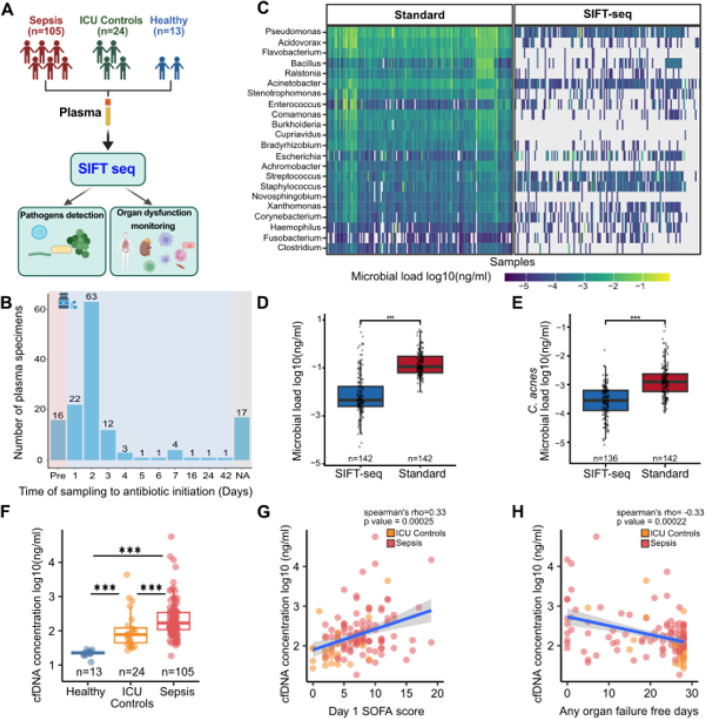
Removal of contaminant DNA in plasma samples of sepsis patients. A) Schematic diagram showing the number of samples included in this study. B) Schematic diagram showing the timing of plasma collection relative to antibiotic therapy initiation. C) Heatmap of abundance of top contaminant genera in standard sequencing vs SIFT-seq. Boxplots of total abundance of microbial DNA originating from D) contaminant genera, E) Cutibacterium acnes in standard sequencing vs SIFT-seq; *Samples where C.acnes microbial load was 0 ng/ul were omitted. F) Comparison of total cell-free DNA concentrations in patient cohorts. G) Correlation of concentrations of cell-free DNA with total day 1 SOFA score. H) Correlation between cell-free DNA concentrations and any organ failure-free days.

We applied a modified version of SIFT-seq (Methods) to 142 plasma samples: 105 from sepsis patients, 24 from non-septic ICU patient controls, and 13 from healthy volunteers (Figure 1A). Of these, 103 Sepsis and 7 ICU control samples were obtained after antibiotics initiation, with most collected between one and two days post treatment (n = 85; Figure 1B, **Tables 1 & 2)**. All blood cultures were performed prior to antibiotic initiation. The median interval between blood culture and plasma collection for sequencing was two days.

### Cell-free DNA abundance is more elevated in Sepsis patients.

The concentration of cell-free DNA (cfDNA) varies with physiological and pathological states^20^ and is elevated in sepsis^21–23^. In our cohort where most plasma samples were collected after antibiotics initiation, cfDNA concentrations were significantly higher in sepsis patients than in ICU controls, and both exceeded those in healthy volunteers (1146.7 ± 5776.1 ng/ml, 334.9 ± 890.6 ng/ml, 22.2 ± 4.17ng/ml for sepsis, ICU controls, and healthy volunteers respectively, Figure 1F). cfDNA levels correlated positively with organ dysfunction, as measured by the SOFA score (Figure 1G, **Spearman’s** rho = 0.33, p-value < 0.05), consistent with prior reports^22,23^. Conversely, cfDNA concentrations correlated negatively with the number of organ failure-free days (Figure 1H, Spearman’s rho = −0.33, p-value < 0.05), a composite outcome reflecting duration of dysfunction while accounting for mortality risk^24,25^. Together, these findings support cfDNA as a biomarker of organ injury in sepsis.

### Removal of Contaminant cfDNA improves sequencing specificity.

The main sources of noise in metagenomic DNA sequencing are misannotation of reference sequences and physical contamination of samples^26,27^. While many strategies address sequence alignment and annotation errors, SIFT-seq was designed to manage physical, environmental DNA contamination. We quantified the abundance of previously reported contaminant genera^26^, using both standard sequencing and SIFT-seq. SIFT-seq markedly reduced contaminant reads: 74% of contaminant genera were eliminated from all samples (Figure 1C). Altogether, we observed an average of 17-fold reduction in abundance of contaminants (8.31 × 10^−5^ ± 1.79 × 10^−4^ ng/ml after standard sequencing, 5.02 × 10^−6^ ± 2.68 × 10^−5^ ng/ml after SIFT-seq, Figure 1D).

We also examined *Cutibacterium acnes*, a common skin commensal and frequent sequencing contaminant, and observed an 11-fold reduction after SIFT-seq (Figure 1E). To further evaluate specificity, we compared the abundance of culture-identified pathogens in culture-positive versus culture-negative samples. For each pathogen, we calculated a signal-to-noise ratio (SNR), defined by the abundance in positive relative to negative cultures(1). When pathogen abundance in culture-negative samples was zero, the SNR was set to zero. SIFT-seq consistently achieved higher SNR values than standard sequencing (median SNR: 80.84 for SIFT-seq vs. 9.55 for standard sequencing; **supplemental Figure S1D**). These findings uphold the improved specificity of SIFT-seq relative to conventional sequencing.

### SIFT-seq enables specific detection of infection-causing pathogens in Sepsis Patients.

**Figure 2:**
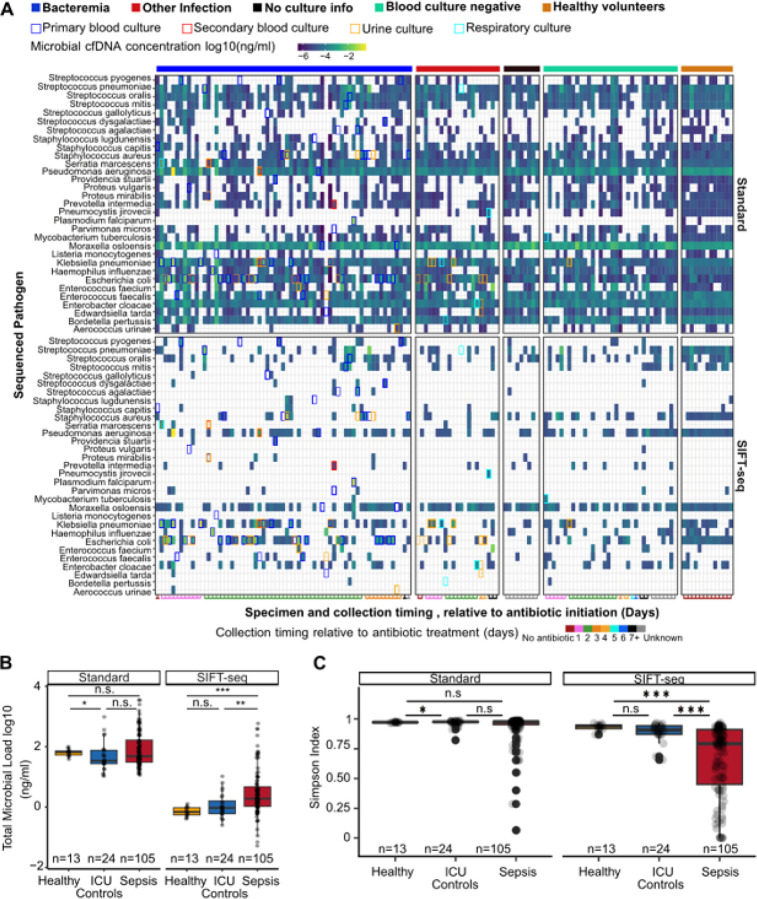
Identification of sepsis-causing pathogens. A) Heatmap showing agreement between SIFT-seq-detected microbes and blood culture, boxes show culture-detected microbes. B) Boxplot showing total microbial load in each patient in all cohorts. C) Box plot showing Simpson diversity index in each patient across all cohorts. Boxes in the box plots indicate the 25th and 75th percentiles, the band in the box indicates the median, and whiskers extend to 1.5 × Interquartile Range (IQR) of the hinge. Outliers (beyond 1.5 × IQR) are plotted individually. *** p-value < 0.001

Microbial cultures collected during or after antibiotic therapy often yield false negatives^28^, whereas metagenomic cfDNA sequencing can detect a broad range of pathogens independent of viability. We therefore compared SIFT-seq with microbial cultures performed before antibiotic treatment initiation (blood: 26 unique species, 63 positive cases; urine: 11 species, 32 cases; respiratory tract: 7 species, 7 cases). We previously demonstrated the superior specificity of SIFT-seq (Figures 1C, D, and E, Figure 2A, **supplemental Figure S1D**) as observed by higher SNR values (80.84 in SIFT-seq vs. 9.55 for standard sequencing), and a significant decrease in background signal compared to the standard metagenomic cfDNA sequencing assay (on average, standard sequencing: 3.67×10^−1^ ± 8.95×10^−1^ ng/ml, SIFT-seq 1.65×10^−1^ ± 7.42×10^−1^ ng/ml). To test SIFT-seq’s sensitivity, we compared microbial cultures to sequencing results. When evaluating the detection rate of pathogens, it is worth noting that microbial cultures were conducted on average two days prior to sample collection for sequencing and before antibiotic initiation.

Of the species detected by culture, SIFT-seq identified microbial cfDNA from 71% of blood culture-confirmed microbes, 71% of respiratory culture-confirmed microbes, and 63% of urine culture-confirmed microbes. Among patients who had already received antibiotics, detection rates were similar (72%, 71%, and 74%, respectively). Standard sequencing showed higher sensitivity (87%, 86%, and 91%) but at the cost of much lower specificity, consistent with its greater susceptibility to contamination. Thus, SIFT-seq achieves sensitivity comparable to conventional cfDNA sequencing while retaining higher specificity, improving discrimination of true pathogens.

Sepsis patients carried significantly higher microbial cfDNA loads than ICU or healthy controls (3.55 × 10^−3^ ± 3.21 × 10^−3^ ng/ml, 5.04 × 10^−3^ ±1.96 × 10^−3^ ng/ml, 0.22 ± 0.86 ng/ml for Healthy, ICU controls, and Sepsis groups respectively, Figure 2B). Microbial load remained elevated in sepsis patients, particularly those with bacteremia, regardless of sampling time after antibiotic initiation (**supplemental Figure S1A–B**).

We next examined microbial diversity. Using the Simpson index, we observed significantly lower diversity in sepsis patients compared to ICU controls after applying SIFT-seq (Figure 2C). This difference was not detected with standard sequencing, underscoring the value of contaminant removal for assessing ecological shifts in the plasma microbiome.

### Immune and Solid-Organ contributions to cfDNA reflect tissue injury in Sepsis.

**Figure 3:**
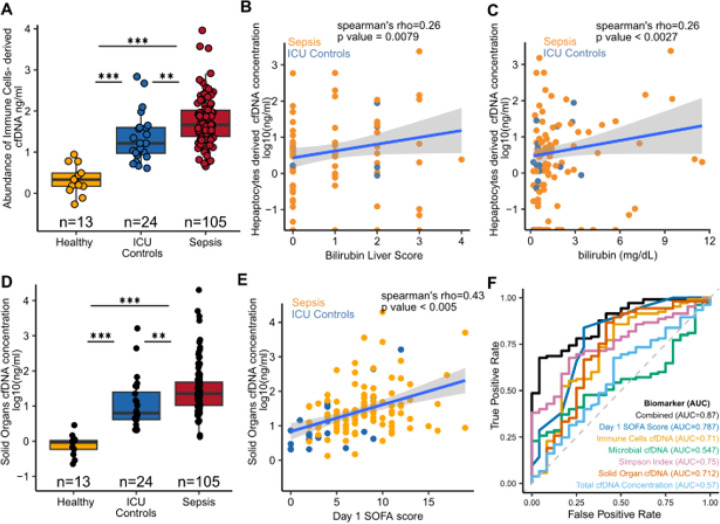
Cell-types of origin of plasma cfDNA and organ damage detection in sepsis patients. A) Boxplot of the abundance of immune cells derived cfDNA. Correlation plots of the amount of hepatocyte-derived cfDNA with B) Bilirubin Liver Score, and C) Bilirubin levels in blood. D) Boxplot of the amount of solid organs-derived cell-free DNA E) Correlation plot of the amount of cell-free DNA originating from cells in solid organs and total day 1 SOFA score F) ROC plot showing the diagnostic capacity of various parameters. Boxes in the boxplots indicate 25th and 75th percentiles, the band in the box indicates the median, and whiskers extend to 1.5 × Interquartile Range (IQR) of the hinge. Outliers (beyond 1.5 × IQR) are plotted individually. *** p-value < 0.001

To assess tissue injury, we deconvolved cfDNA methylation profiles using a reference atlas spanning 40 cell types across multiple organ systems^29^. Sepsis patients showed a marked increase in immune cell-derived cfDNA compared to controls, with granulocytes as the dominant contributor, followed by macrophages, monocytes, and megakaryocytes (Figure 3A). Together, these accounted for more than half of host cfDNA in sepsis patients. Smaller but detectable contributions came from hepatocytes, endothelial cells, and other solid-organ cell types (**supplemental Figure S1F**).

Consistent with prior reports^22,23^ of hepatic injury in sepsis, liver-derived cfDNA was significantly elevated in sepsis patients relative to controls (**supplemental Figure S1E**). Liver cfDNA levels correlated with both serum bilirubin (Spearman’s rho = 0.27, p-value = 0.0026, Figure 3C) and bilirubin SOFA score (Spearman’s rho = 0.26, p-value = 0.0079, Figure 3B), which are used in the diagnosis of liver function in sepsis.

To further resolve cell-type contributions, we quantified the total amount of cfDNA derived from solid organs, in other words, cfDNA originating from sources other than blood and lymphatic system. Solid organ-derived cfDNA was significantly higher in sepsis patients than in other groups (Figure 3D) and correlated with total day-1 SOFA score (Spearman’s rho = 0.26, p = 0.0027; Figure 3E). These findings indicate that cfDNA profiling can capture both immune activation and organ-specific injury during sepsis.

### Comparison of the Diagnostic Potential of Host- and Microbe-Derived cfDNA Metrics to the Total Day 1 SOFA Score.

A key challenge in critical care is distinguishing sepsis from noninfectious inflammatory conditions. We therefore evaluated the diagnostic performance of cfDNA-derived metrics and composite scores, including cfDNA concentration, microbial load, Simpson diversity index, immune cell-derived cfDNA, and solid organ-derived cfDNA, relative to the total day-1 SOFA score.

We again note that most plasma samples were collected after antibiotic initiation. Despite this, receiver operating characteristic (ROC) analysis demonstrated that individual cfDNA-derived parameters had slightly lower, yet comparable, diagnostic performance relative to the SOFA score (Figure 3F, **Table 3)**. Among the cfDNA metrics, the Simpson Index yielded the highest performance (AUC = 0.75; 95% CI: 0.675–0.855), closely approaching that of the SOFA score (AUC = 0.787; 95% CI: 0.673–0.901). We then asked whether combining host- and microbe-derived cfDNA features with SOFA could improve discrimination. Indeed, a multivariate logistic regression model incorporating all cfDNA metrics plus SOFA achieved the highest accuracy (AUC = 0.874; 95% CI: 0.803–0.945), surpassing any single parameter.

## DISCUSSION

In this study, we evaluated metagenomic cfDNA analysis via SIFT-seq to identify sepsis-causing pathogens during antibiotic therapy and to simultaneously assess organ injury and host response. We show that SIFT-seq detects sepsis-causing pathogens in the plasma cfDNA of these patients with a sensitivity comparable to cultures and conventional metagenomic DNA sequencing^30–33^, while achieving improved specificity. Plasma cfDNA also captured pathogens detected in urine and respiratory cultures, underscoring its potential value beyond blood-based testing. The absence of some culture-identified pathogens in both sequencing methods likely reflects sample timing: sequencing was performed a median of two days after cultures, often after antibiotics had been started. Our analysis was also limited to bacterial and DNA viral pathogens, as RNA viruses and fungi were excluded from the reference database.

SIFT-seq revealed increased microbial load and reduced microbial diversity in sepsis patients compared to ICU and healthy controls. These patterns were obscured in conventional sequencing because of contamination-derived background. The lack of association between microbial cfDNA abundance and duration of antibiotic therapy was unexpected but may be confounded by patient heterogeneity^34–37^. Larger longitudinal studies will be needed to define the kinetics of pathogen cfDNA during treatment.

Methylation-based deconvolution of cfDNA confirmed prior reports^33,38,39^, which were conducted with smaller sample sizes, that granulocytes are the major contributors to cfDNA in sepsis.^39^ Elevated cfDNA from hepatocytes and other solid organs was associated with baseline organ dysfunction, supporting cfDNA as a potential marker of tissue injury. Increased immune cell-derived cfDNA further suggests that cell death contributes to the dysregulated host response^22,23,35,40^. More recently, non-apoptotic programmed cell death mechanisms, such as necroptosis, pyroptosis, and neutrophil extracellular trap (NET)- associated cell death (NETosis), have been implicated in the pathogenesis of sepsis^41,42^. Elevated neutrophil-derived cfDNA in our cohort is potentially consistent with NETosis, though future work should correlate these signals with independent NET biomarkers^43^.

This analysis has several key strengths. The large patient cohort is derived from a well-phenotyped and carefully adjudicated patient population. Additionally, despite being derived from a single center, the range of unique pathogens is extensive, including *Pneumocystis jirovecii*, *Plasmodium falciparum*, and *Staphylococcus aureus*. Moreover, the distribution of organ dysfunction within this population is broad and largely representative of sepsis in the ICU. Weaknesses of this analysis include the lack of serial samples to make within-patient comparisons that could establish the potential role of quantitative microbial cfDNA in the monitoring of potential treatment. However, sepsis and infections more broadly have no clear “time zero” and the presentation to medical care is often stochastic. Future work including samples collected prior to antimicrobial treatment and at follow-up have the potential to answer additional questions about the sensitivity and kinetics of microbial cfDNA. We were unable to quantitatively detect many tissue-specific subtypes of cfDNA in the current analysis despite extant multisystem organ failure. This raises the possibility that certain organ failures are not accompanied by readily detectable circulating cfDNA from the same failing organ. It is possible, however, that organ dysfunction may not be accompanied by significant parenchymal cell death^44^. Whether amounts below the limit of our methodology are present in the circulation is unknown.

Taken together, these results provide support for the potential of SIFT-seq as a comprehensive diagnostic tool, capable of detecting sepsis-causing pathogens with high sensitivity and specificity, even after antimicrobial therapy, while concurrently profiling organ injury from minimal plasma input.

## METHODS

### Study Cohort and sample collection.

Since 2014, investigators have prospectively consented to patients admitted to any ICU at NYP-WCMC to participate in a registry involving the collection of biospecimens and clinical data^45^. For each participant, whole blood (6–10 ml) was obtained. Whole blood samples were drawn into EDTA-coated blood collection tubes (BD Pharmingen, San Jose, CA). Samples were stored at 4°C and centrifuged within 4 hours of collection. Plasma was separated and divided into aliquots and kept at −80°C. The registry was approved by the institutional review board of WCMC (1405015116, 20–05022072). Patients with a clinical diagnosis of sepsis, details below, are the main analytic population. Patients from the same registry without a concern for infection were used as the ICU control population. Healthy controls were derived from healthy volunteers recruited for blood donations through a protocol approved by the Cornell Institutional Review Board (protocol number 1910009101).

### Sepsis definitions.

Clinical and laboratory data were collected from the EHR at NYP-WCMC by trained research personnel. Organ failure was defined by the SOFA scoring system^6^. Missing individual organ system scores were designated as 0. Patients in the sepsis group had a clinically documented or suspected infection that was adjudicated as the primary source of organ dysfunction. Clinical adjudication of the final diagnosis of sepsis was confirmed by board–certified critical care physicians.

### SIFT-seq in plasma.

An aliquot of 520 μL of plasma was centrifuged at 20,000 × g (~14,000 RPM) for 10 minutes at 12°C to pellet cellular debris. The supernatant was transferred to a new 1.5 ml tube, and the final volume was brought up to 1000 μL with PBS. The solution was heated to 98°C for 10 minutes and mixed at 190 × g(1000 RPM) to coagulate the albumin present in plasma. The solution was then centrifuged at 1600 xg (~4000 RPM) for 10 minutes. 500 μL of supernatant was transferred to a 15 Falconcon tube containing 3.25 ml of ammonium bisulfite solution (Zymo Research, product #5030) and shaken in a thermomixer at 98°C for 10 minutes (15s on/30s off). Samples were then transferred to a thermomixer at 54 °C for 60 minutes (15s on/30s off). Then, cfDNA extraction was performed using the QIAamp Circulating Nucleic Acid Kit using the 4-ml plasma protocol (Qiagen, product #55114). Prior to DNA elution, 200 μL of L-Desulphonation buffer (Zymo Research, product #5030) was added to the columns for 15 minutes, followed by two washes with 200 μL absolute ethanol. DNA was then eluted according to manufacturer recommendations, and single-stranded library preparation was performed (Claret Biosciences, product #CBS-K150B). Libraries were then sequenced on an Illumina sequencer. A step-by-step protocol is provided in the supplementary information file.

### Sequencing Library Preparation.

Bisulfite conversion of cfDNA involves a cfDNA denaturing step at 98°C, resulting in single-stranded cfDNA molecules after DNA extraction. For this reason, a single-stranded sequencing library preparation method is chosen for the next steps. We prepared sequencing libraries using the SRSLY PicoPlus DNA NGS Library Preparation Base Kit (SRSLY Cat# CBS-K250B-24) with the SRSLY UDI Primer Set-24 (SRSLY Cat# CBS-UD-24) following the manufacturer’s protocol, with the following modifications:

1. The input cfDNA volume used was 18 μL.
2. 1.25 μL of NGS Adapters A and 1.25 μL of NGS Adapters B were added to the 20 μL denatured DNA reaction tube, and the volume was completedby 1.5 μL of ultrapure water.
3. The Index PCR Master Mix was substituted for an equal volume of KAPA HiFi Uracil+ Ready Mix (2X).
4. The Indexed Library DNA Purification step was performed twice, first eluting in 50 μL and then in 25 μL.

### Alignment to the human genome.

Adapter and low-quality bases from the reads were trimmed using BBDuk (BBDuk V38.46^46^, --entropy= ‘0.25’ --maq= ‘10’ -Xmx1g tbo tpe) and aligned to the C-to-T and G-to-A converted human genome using Bismark (Bismark-0.22.1^47^, --unmapped, --quiet). PCR duplicates were removed using Bismark.

### Depth of coverage.

The depth of sequencing was measured by summing the depth of coverage for each mapped base pair on the human genome after duplicate removal, and dividing by the total length of the human genome (hg19, without unknown bases).

### Removing unconverted molecules.

Aligned BAM files are filtered to remove unconverted molecules using the Bismark^47^ (Bismark-0.22.1) alignment package with default parameters.

### Bisulfite conversion efficiency.

We estimated bisulfite conversion efficiency by quantifying the rate of C[A/T/C] methylation in human-aligned reads (using MethPipe V3.4.3^48^), which are rarely methylated in mammalian genomes.

### Pre-processing of the unmapped reads.

Reads originating from the Phix genome were removed from the host unmapped reads using Bowtie 2^49^ (Bowtie 2.4.3, --local, --very-sensitive-local, --un-conc). Read IDs from the remaining reads were used to subset paired-end reads from the original FASTQ files. Adapter trimming and read quality filtering were performed using BBDuk^46^ (BBDuk V38.46, maq=32). Remaining reads were deduplicated using samtools^50^ (samtools V1.14) and merged using FLASH2^51^ (-q -M75 -O). K-mer decontamination to remove human reads was then performed using BBDuk^46^ (BBDuk V38.46, k=50, prealloc = t), and the obtained fastq file was converted to a fasta file for metagenomics analysis.

### Metagenomic abundance estimation from sequencing data.

Reads mapping to microbial species were identified using HS-BLASTN^52^ (hs-blastn-1.0.0), and microbial abundances were estimated using GRAMMy (version 1)^53^. Specific to SIFT-seq, read-level filtering of contaminants is performed by removing sequenced reads with 4 or more cytosines present, or one methylated CpG dinucleotide (the latter represents unmapped, human-derived molecules). Species-level filtering based on the distribution of mapped reads is carried out by first aligning filtered and unfiltered datasets independently. Cytosine densities of mapping-coordinates in both datasets are measured using custom scripts, and their distributions are compared using a Kolmogorov-Smirnov test. Significantly different filtered-unfiltered distributions are further processed (D-statistic > 0.1 and p-value < 0.01). Briefly, filtered datasets whose distribution of cytosines at mapped locations is significantly lower than unfiltered datasets have one read removed and are tested for differences in their distribution. If the distributions are more similar (as measured through the same criteria), it is filtered out. This process is repeated until distributions are no longer significantly different, or if all reads are removed. Read and species-level filtering were performed using custom scripts written in Python. Microbial abundance in downstream analyses was quantified as Molecules Per Million reads (MPM).

### Statistics and reproducibility.

All statistical methods were performed in R version 4.0.5. Groups were compared using two-sided Wilcoxon Signed Rank or Wilcoxon Rank Sum tests. Boxes in the boxplots indicate 25th and 75th percentiles, the band in the box indicates the median, and whiskers extend to 1.5 × Interquartile Range (IQR) of the hinge.

**Signal-to-Noise Ratio(SNR)** per species was calculated using the following equation:

Equation (1) $$\text{SN}R_{\text{species}}=\frac{\text{Mean}\;\text{Abundance}\;\text{in}\;\text{culture}\;\text{positive}\;\text{specimens}\;}{\;\text{Mean}\;\text{Abundance}\;\text{in}\;\text{culture}\;\text{negative}\;\text{specimens}\;}$$

In cases where the median abundance for culture-negative specimens was null, we equated the Signal-to-Noise Ratio to 0.

Investigators were blinded to group allocation during data collection of samples in the Sepsis cohort. Groups and detailed clinical information (e.g., data from conventional blood cultures) were shared with the investigators after the data were analyzed and shared with collaborators, who then shared metadata elements. Experiments were not randomized.

## Supplementary Files

This is a list of supplementary files associated with this preprint. Click to download.

- SupplementaryInformation.docx

## Data Availability

Sequencing data from human plasma cfDNA is available in the database of Genotypes and Phenotypes (dbGaP), accession number phs001564.v1.p1.
